# A literature review on the parvovirus B19 infection in sickle cell anemia and β-thalassemia patients

**DOI:** 10.1186/s41182-020-00284-x

**Published:** 2020-12-02

**Authors:** Saber Soltani, Armin Zakeri, Alireza Tabibzadeh, Milad Zandi, Elham Ershadi, Sara Akhavan Rezayat, Sanaz Khaseb, Amir mohammad Zakeri, Mohammadvala Ashtar Nakhaei, Shervin Afzali, Abbas Farahani

**Affiliations:** 1 Department of Virology, School of Public Health, Tehran University of Medical Sciences, Tehran, Iran; 2 Department of Hematology, Faculty of Medical Sciences, Tarbiat Modares University, Tehran, Iran; 3 Department of Virology, Iran University of Medical Sciences, Tehran, Iran; 4 Department of Health Care Management and Economics, School of Public Health, Tehran University of Medical Sciences, Tehran, Iran; 5 Pediatric Surgery Research Center, Research Institute for Children’s Health, Shahid Beheshti University of Medical Sciences, Tehran, Iran; 6 Department of Cellular and Molecular Biology, Faculty of Life Sciences and Biotechnology, Shahid Beheshti University G.C, Tehran, Iran; 7 Infectious and Tropical Diseases Research Center, Hormozgan Health Institute, Hormozgan University of Medical Sciences, Bandar Abbas, Iran

**Keywords:** Parvovirus B19, Sickle cell anemia, β-thalassemia

## Abstract

**Background:**

Parvovirus B19 is the causative agent for erythema infectiosum, and also as a potentially life-threatening infectious agent, it is mainly presented in high erythrocyte turnover patients. Sickle cell disease (SCD) is an inherited monogenic hematological disorder resulting from the mutations in the hemoglobin β-chain gene. Thalassemia is a hereditary hematological syndrome that happens in consequence of deficiencies in the production of one or more globin chains. We summarize current knowledge about the prevalence rates of the parvovirus B19 infection in sickle cell anemia and thalassemia patients.

**Methods:**

Several online databases were searched including, Scopus, EMBASE, Web of Science, Google Scholar, and PubMed, which were performed amidst 2009–2019 by using distinct keywords: “Thalassemia,” “Parvovirus,” “Anemia,” “Sickle cell anemia,” “parvoviridae,” “parvoviridae infection,” and “parvovirus B19.”

**Results:**

Search results indicated 4 and 7 studies for the prevalence of the parvovirus B19 in β-thalassemia and SCD, respectively. Among the β-thalassemia patients, the B19V seroprevalence for IgG and IgM were ranged from 18.2–81% and 14.5–41.1%, respectively; meanwhile, B19V DNA positively results was 4–15.3%. Moreover, in the SCD group, the extent of B19V IgG was varied from 37.6 to 65.9% and that of IgM was in a range of 2.9–30%, and the DNA detection rate was 4–54%.

**Conclusion:**

B19V seroprevalence changes in several conditions including, different epidemiological features, socio-economic status, and overpopulation. Age can expand the incidence of anti-B19V IgG/IgM in SCD and beta-thalassemia patients. Reinfection and diverse genotypes are relevant factors in the seroprevalence of B19v. The patients’ immunological-hematological station and higher abundance of transfusions can affect the B19V seroprevalence in SCD and beta-thalassemia group. Further investigations in this field could be suggested to better understand the virus distribution in this susceptible population of patients.

## Introduction

The *Parvoviridae* family comprises two subfamilies named *Densovirinae* and *Parvovirinae*; the latter afflicts vertebrates, and the *Erythrovirus* genus, and parvovirus B19 are its significant members [1]. The human parvovirus B19 is a small, linear, and single-stranded DNA virus. The unexpected discovery of this virus was first reported by Cossart et al. in 1975; it was found when the donor sera were being tested for hepatitis B virus [2, 3]. It is mentioned that respiratory droplets are the means for the transmission of the B19 virus, and close contacts like household contact are also the other possible ways [4, 5]. B19 can pass through the placenta and infect the baby. Moreover, it is likely to be transmitted through blood transfusions [6, 7].

Sickle cell disease (SCD) is an inherited monogenic hematological disorder. Homozygous missense mutations in the hemoglobin β-chain gene lead to the generation of sickle hemoglobin (HbS), and it is also responsible for chronic damage to various organs [8, 9]. These mutations lead to thymine and glutamic acid substitution to adenine and valine, respectively [10]. In hypoxemia condition, HbS reduces the flexibility of RBCs (red blood cells) [11]. As a result, these fragile RBCs will increase the hemolysis and lead to chronic anemia [9].

Furthermore, sickled erythrocytes disrupt the blood flow in small capillaries and lead to a variety of disorders [11]. Thalassemia is a hereditary hematological syndrome with deficits in the production of one or more globin chains. The clinical characterizations are diverse, ranging from hypochromia and microcytosis to hemolytic anemia. The absence or reduction of β-globin chains happens in β-thalassemia patients [12, 13]. Although major β-thalassemia patients need regular blood transfusions, it might cause a higher risk for B19 infection through blood transfusions [14–16]. Furthermore, B19 is thought to be a potentially life-threatening infectious agent in patients with high erythrocyte turnover [17]. By and large, this paper explores the prevalence rates of parvovirus B19 infection in sickle cell anemia and thalassemia patients based on the study inclusion criteria.

## Methods

### Study design and search strategy

This systematic review was carried out based on the criteria drafted in the Preferred Reporting Items for Systematic Reviews and Meta-Analysis (PRISMA) statement [18]. The PRISMA checklist was completed in all steps of this research. We did not register the study protocol before the initiation of the study.

**Fig. 1:**
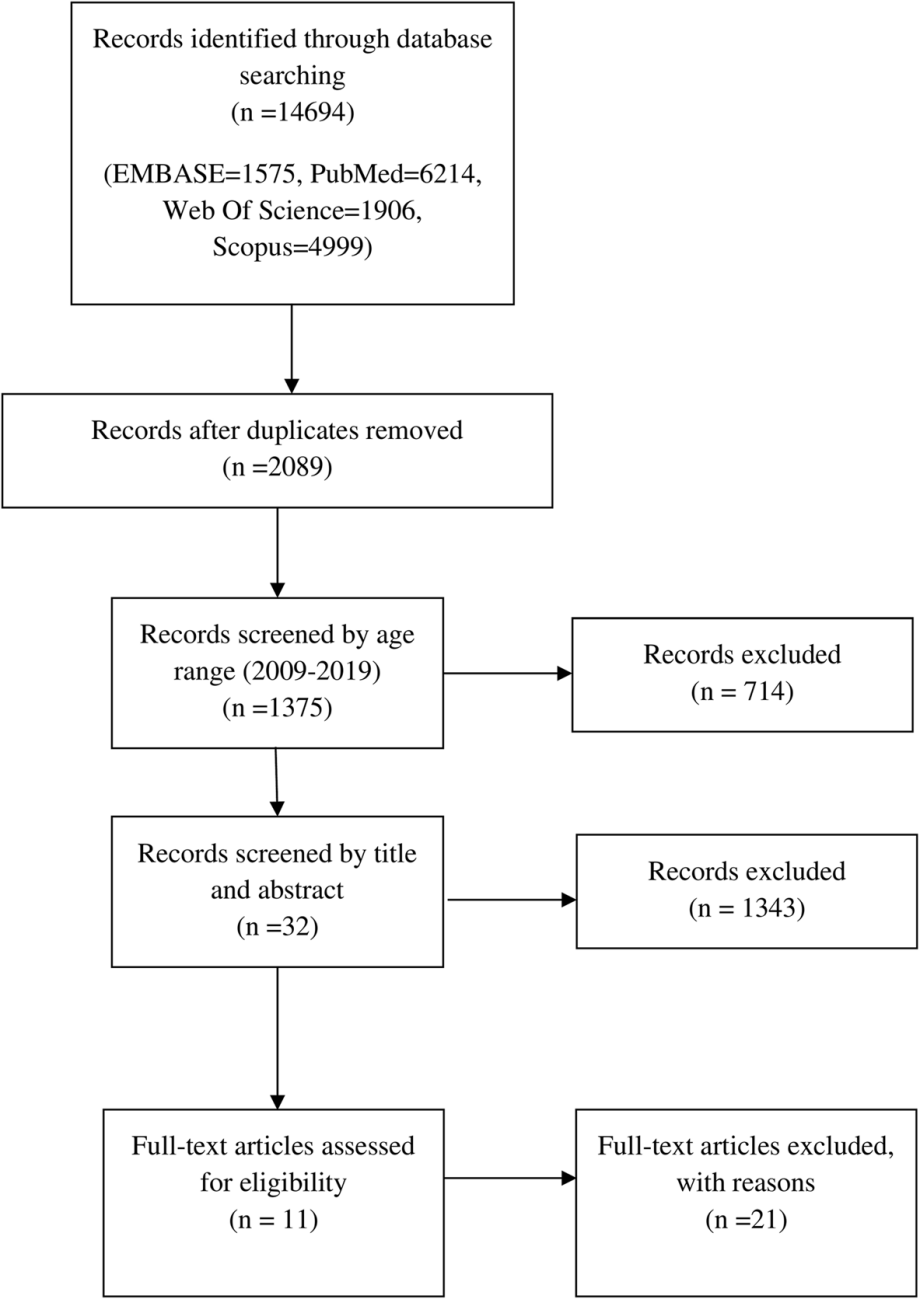
Conducted study search flowchart

In the current study, a literature search was performed in the English language from 1 January 2009 until 2019. The search process was performed in electronic databases, such as PubMed, Scopus, Web of Science, and Embase. For the search processing in the current study according to medical subject headings (MeSH), the following keywords were used “Thalassemia,” “Parvovirus,” “Anemia,” “Sickle Cell anemia,” “parvoviridae,” “parvoviridae infection,” “parvovirus B19.” The reports were reviewed and managed with EndNote X8 (Thomson Reuters). The search was implemented by two independent researchers, and a third researcher checked the findings (Fig. 1).

### Inclusion and exclusion criteria

The conducted search results were imported in EndNote Version X8 (Thomson Reuters), and the duplicate studies were removed. The screening was done according to the title and the abstract of articles, and finally, the full-text was assessed. The administered inclusion criteria were as follows: (1) investigations in cross-sectional, cohort, and case-series design that monitored the B19 infection in sickle cell anemia and β-thalassemia patients (2) articles published in English language and between 2009 and 2019 and (3) observational studies were also included. All included articles met the inclusion criteria. Absence of significance and relevance of the data in the articles and the ones that did not meet the eligibility criteria were excluded. The non-representativeness of the study population and those studies utilized of non-standard estimation devices were also removed. Moreover, case reports and case series with no significant sample size were excluded. These review articles (the literature reviews which were screened for relevant data), congress abstracts, and letters to the editor were excluded.

### Qualitative assessment

All the eligible studies entered were evaluated for risk of bias utilizing the Critical Appraisal Skills Programme (CASP) checklist. The risk of bias in particular studies was ranked as low, moderate, and high. Studies with a high risk of bias were omitted.

### Data extraction

To prevent bias, two independent reviewers double-screened the final search results based on titles, abstracts, and full texts of articles according to inclusion and exclusion criteria. The third expert researcher solved the conflicts. The data of all included studies were summarized by using a data extraction sheet in EXCEL software, which covered the first author’s name, country, publication year, thalassemia or sickle cell disease patients, thalassemia type, sample size, age, sex, IgG, and IgM prevalence, B19 virus genotypes, serological (ELISA), and molecular (PCR) diagnostic methods and study design.

## Results

In this literature review, the results of eleven different studies that assessed the parvovirus B19 infection in two different groups of patients were compiled. The conducted search result is illustrated in Fig. 1. Also, four studies evaluated general factors in patients with thalassemia. Six studies focused on patients with SCD. One study assessed parvovirus in patients with either thalassemia or sickle cell disease. Overall, 334 thalassemia and 1016 SCD patients were identified in these articles. The results were summarized in Tables 1 and 2. The following studies were published between 2011 and 2017.

**Table 1:** Parvovirus B19 prevalence in β-thalassemia patients

First author	Country	Year	Sample size		Mean age (year)	Sex^a^		IgG prevalence (%)		IgM prevalence (%)		Genotype	PCR prevalence	Method	Study design	Ref.
			Case	Control		Male	Female	Case	Control	Case	Control					
Ghwass	Egypt	2016	55		6.2± 13.25	29	26	18.2		14.5		–	–	ELISA	CS	[19]
Slavov	Brazil	2012	39	100	23.56 (range: 3–45)	59%	41%	35.9	60	–		1A	15.3	sequencing, ELISA, qPCR,	CS	[20]
Kishore	India	2011	90	32	8 (range 2-18)	55	35	81	21	41.1	6.2	–	–	ELISA	CS	[21]
Arabzadeh	Iran	2017	150		30.4	75	75	–		–		I	4	Real-time PCR	CS	[22]

^a^Some studies reported the number of male/female and another reported percentage

*PCR* polymerase chain reaction, *CS* cross-sectional, *ELISA* enzyme-linked immunosorbent assay, *qPCR* quantitative PCR

**Table 2:** Parvovirus B19 prevalence in SCD patients

First Author	Country	Year	Sample size		Mean age (year)	Sex^a^		IgG prevalence (%)		IgM prevalence (%)		Genotype	PCR prevalence		Method	Study Design	Ref.
			Case	Control		Male	Female	Case	Control	Case	Control		Case	Control			
Slavov	Brazil	2012	144	100	19.22 years (range: 1–72 years)	48.6%	51.4%	65.9	60	–		1A	19.4	1	Sequencing, ELISA, qPCR,	CS	[20]
Ayolabi	Nigeria	2017	68	25	1–35 years	44	49	–		15	16	–			ELISA	CS	[23]
Obeid	Saudi Arabia	2011	138	56	28	77	61	37.6	39.3	2.9		–	4		ELISA, real-time PCR	CS	[24]
Makhlouf	Egypt	2015	100	60	7.8 ± 1.7	50	50	44	10	30	0	–	54	3.3	Nested-PCR, ELISA	CS	[25]
Hankins	USA	2016	330		7.6 (range 0.4–18)	–		38		–		–	–		ELISA	CS	[26]
Diallo	Africa	2011	163	163	1–45 years	91	72	64.8	48.4	25	6.1	–	–		ELISA	CC	[27]
Iwalokun	Nigeria	2013	73	81	16.9 ± 0.7	40	33	61.6	64.2	17.8	11.1	–	11.1		ELISA, PCR	CS	[28]

^a^Some studies reported the number of male/Female and another reported percentage

*CC* case-control, *PCR* polymerase chain reaction, *CS* cross-sectional, *ELISA* enzyme-linked immunosorbent assay, *qPCR* quantitative PCR

### Parvovirus B19 estimated prevalence in beta-thalassemia major patients

Among four studies focused on the prevalence of the parvovirus B19 in β-thalassemia, beta-thalassemia patients’ lowest mean age was 6.2 ± 13.25 years in Egypt, and the highest mean age degree was 30.4 years in Iran. However, considering the mean age result between beta-thalassemia patients, most cases were in childhood. The B19V seroprevalence is dependent on age, and it rises steadily from early childhood to the elderly. Presumably, some of the included studies in our review, from the India Province, showed an association between the prevalence of parvovirus B19 infection and several factors such as childhood age, poor socio-economic conditions, and overpopulation.

Taking these four studies into account, it is noteworthy that in 184 patients, the anti-B19V IgG in β-thalassemia patients ranged from 18.2 to 81%. The result of three different studies indicates that the anti-B19V IgM in β-thalassemia patients ranged from 14.5 to 41.1% in 145. Furthermore, the B19V DNA predominance rate was shown to be 4–15.3% in 189 patients.

### Parvovirus B19 prevalence in SCD patients

Seven different studies focused on the prevalence of the parvovirus B19 in SCD were taken into consideration. The mean age of SCD patients showed different ranges in economically developing countries such as Nigeria (16.9 ± 0.7 years) and rich countries like Saudi Arabia, i.e., 28 years.

### Anti-B19V IgM

In six different studies, the anti-B19V IgG levels in SCD patients’ sera was varied from 37.6 to 65.9% in 948 patients. Furthermore, in five different studies, it was indicated that the anti-B19V IgM in SCD patients ranged from 2.9 to 30% in 542 patients. The parvovirus B19 DNA predominance rate was 4–54% in 455 patients in four separate studies.

## Discussion

Human parvovirus B19, the causative agent for erythema infectiosum (fifth disease), has a tropism for erythroid progenitor cells, and in consequence, it can temporarily suppress erythropoiesis in bone marrow [29, 30]. In SCD and Thalassemia patients, the parvovirus B19 infection makes more severe conditions, known as a transient aplastic crisis [20, 31]. The β-thalassemia patients have a higher risk for the infection of the parvovirus B19 due to the necessity of multiple blood transfusions throughout their life [32]. In the study carried out by Kishore et al. in northern India, the frequency of parvovirus B19 infection was assessed in 90 major β-thalassemia patients with a hyper-transfusion regimen who had 10–360 units of blood transfusion in their whole life and 32 controls. The results of the study indicated that the age range of participants was 2–18 years, and 61% were males. In house ELISA for VP-1 and VP-2 was used to detect anti-B19 IgM/IgG antibodies; it revealed that the parvovirus IgG and IgM prevalence in major β-thalassemia patients were 81% and 41%, respectively. It was also suggested that the seroprevalence of the parvovirus infection and the numbers of blood transfusion times are directly proportional [21]. Furthermore, Arabzadeh and colleagues investigated blood samples of 150 β-thalassemia major patients (75 males, 75 females) by real-time PCR to determine the prevalence of parvovirus B19 DNA. This study's results indicate that 4% of enrolled patients were positive for parvovirus B19 DNA, and half of these participants were aged 26–30 years and others were 31–35 [22].

The academic community has extensively explored the titer of B19 IgG and IgM in SCD patients. Having said that, Ayolabi et al. investigated the level of parvovirus B19 IgM antibody in 68 SCD patients and 25 control samples by ELISA. Anti-parvovirus B19 IgM was detected in 15% of SCD patients. Also, Obeid assessed the parvovirus B19 antibody and DNA in 138 SCD patients. Obeid’s study showed that the parvovirus B19 IgG and IgM prevalences are 37.6% and 2.89%, respectively. Moreover, the parvovirus B19 DNA was detected in all IgM positive patients [24]. In another study in Egypt, Makhlouf et al. assessed the serological and molecular prevalence of the parvovirus B19 in 100 SCD patients and 60 controls. The results revealed that 30% of SCD patients were positive for the parvovirus B19 IgM and DNA, while 24% had positive IgG and DNA by nested-PCR [25].

Parvovirus B19 seroprevalence alters in various countries due to different causes. Hankins et al. reported that the incidence of parvovirus B19- specific antibody responses in pediatric SCD cases is expanded with age, as suspected due to an increased likelihood of parvovirus B19 exposure [26]. In the Arabzadeh et al. study, parvovirus-infected patients’ mean age was 30.4 years [22]. The epidemiological aspects can affect the B19V seroprevalence in different groups of patients. Although this epidemiological division shows the immune response to B19V, it confirms the risen viral dissemination between elderly patients with SCD [20]. Reinfection is another relevant factor in maintaining B19V seroprevalence [20]. The different genotypes can influence antibody-positive and DNA-positive samples in different countries of Europe and Africa [24]. Kishore et al. reported no significant associations between parvovirus prevalence and thalassemia patients' age and gender. They mentioned that the B19V seroprevalence was raised to the low socio-economic status or a higher abundance of transfusions received by these patients [21]. Prior investigations queried the factors impacting on its seroprevalence. Ghwass et al. stated that the differences in B19V seroprevalence in different areas are dependant on the climate, variable socio-economic status, overpopulation, geographical, and the variety in the immunological-hematological status of patients [19].

The differences in all these studies could be due to the differences in the sample size and geographical distribution. This study was conducted as a preliminary study that could be useful for further systematic review and meta-analysis by narrowing it in this field of study. It could be used entirely as a reminder for more protective actions about parvovirus B19 transmission in susceptible patients. The drawback of this framework was the absence of quantitative analysis

## Conclusion

Parvovirus B19 seroprevalence changes in several conditions in different countries and communities. The age can expand the incidence of anti-B19V IgG/IgM in SCD and beta-thalassemia patients. Also, the epidemiological features can affect the B19V seroprevalence in groups of patients. Reinfection and diverse genotypes are additional relevant criteria in maintaining B19V seroprevalence and DNA-positively results. However, the differences in B19V seroprevalence in different areas are dependent on the socio-economic situation and overpopulation. Additionally, the difference in patients' immunological-hematological condition and higher abundance of transfusions received by patients are other factors that affect the B19V seroprevalence. Further investigations in the field of SCD or thalassemia and B19V infection is suggested to gain a better understanding of the virus distribution in susceptible populations.

## Data Availability

All data associated with this manuscript is inclusive in this paper.

## Abbreviations

SCD: Sickle cell disease
IgG: Immunoglobulin G
IgM: Immunoglobulin M
B19V: Parvovirus B19
RBCs: Red blood cells
PCR: Polymerase chain reaction
CS: Cross-sectional
CC: Case-control
ELISA: Enzyme-linked immunosorbent assay
qPCR: Quantitative PCR
VP: Viral protein
